# Chlorpyrifos Toxicity in Mouse Cultured Cerebellar Granule Neurons at Different Stages of Development: Additive Effect on Glutamate-Induced Excitotoxicity 

**DOI:** 10.22074/cellj.2016.4575

**Published:** 2016-08-24

**Authors:** Nahid Amani, Maliheh Soodi, Bahram Daraei, Abolfazl Dashti

**Affiliations:** Department of Toxicology, Faculty of Medical Sciences, Tarbiat Modares University, Tehran, Iran

**Keywords:** Chlorpyrifos, Neurotoxicity, Glutamate Toxicity, CGNs, Oxidative Stress

## Abstract

**Objective:**

Chlorpyrifos (CPF) is a neurotoxic organophosphorus (OP) insecticide. Its mechanism of action includes oxidative stress, excitotoxicity, and inhibition of the acetylcholinesterase enzyme (AChE). The aim of the present study is to investigate CPF toxicity in mature and immature cerebellar granule neurons (CGNs), as well as its effect on glutamate induced excitotoxicity.

**Materials and Methods:**

This study was an *in vitro* experimental study performed on mice cultured CGNs. Immature and mature neurons were exposed to different concentrations of CPF (1-1000 µM) and glutamate (10-600 µM) for 48 hours after which we used the MTT assay to measure cytotoxicity. Immature neurons had exposure to CPF for 5 days in order to evaluate the cytotoxic effect on developing neurons. Mature neurons received sub-lethal concentrations of CPF (10, 100 µM) combined with different concentrations of glutamate. AChE activity and reactive oxygen species (ROS) generation were assessed after treatments.

**Results:**

Immature CGNs had increased sensitivity to CPF toxicity compared to mature neurons. We observed significantly greater ROS production in immature compared to mature neurons, however AChE activity was more inhibited in mature neurons. Although CPF toxicity was not well correlated with AChE inhibition, it correlated well with ROS production. Glutamate toxicity was potentiated by sub-lethal concentration of CPF, however glutamate induced ROS production was not affected. The results suggested that CPF potentiated glutamate toxicity by mechanisms other than oxidative stress.

**Conclusion:**

CPF toxicity differed in mature and immature neurons. Potentiated glutamate toxicity by CPF implied that CPF exposure might be a risk factor for neurodegenerative disease.

## Introduction

The most well-known organophosphorus (OP) insecticide is chlorpyrifos (CPF) which is widely used because of its great stability, relative safety and broad spectrum of domestic and agricultural applications compared to other OP pesticides (1). Widespread use of CPF is concerning to public health. Long-term low dose effects of OP pesticide exposure are linked to cancers, diabetes, and a wide range of neurological disorders such as depression and neurodegenerative disease. However the mechanism of the effect of OP on the pathophysiology of these diseases is still unknown (2,4). 

The main toxicity of CPF is related to irreversible inhibition of acetylcholinesterase (AChE) in central cholinergic synapses (5). It has been assumed that neurologic disorders following low level exposure to CPF result from the inhibitory effect of AChE (6). However, recent concern focuses on another mechanism of CPF neurotoxicity that is independent of AChE inhibition (7). *In vitro* studies indicate that some non-AChE targets are involved in CPF neurotoxicity. CPF can cause neural death by inducing apoptosis and mitochondrial dysfunction (7,8), by direct interaction with the cholinergic system, and/or modulation of receptor expression. CPF can decrease α7nAChE expression (9) and block α4β2-mediated currents (10). This insecticide and its metabolites disrupt cAMP production which is involved in muscarinic receptor signaling (11). It is clearly understood that CPF can cause increased production of reactive oxygen species (ROS) and oxidative stress in cells, especially in neurons. CPF has been reported to cause dose-dependent neuronal death among cultured hippocampal neurons (12). Recently, mice exposed to CPF (30 mg/ kg) had evidence of dysregulated dopamine signaling and glutamatergic neurotransmission (13). In addition, CPF has been reported to increase glutamate release and induce glutamate-mediated excitotoxicity in a cortical neuron culture (14). 

Excitotoxicity is defined as cell death that results from the toxic actions of excitatory amino acids. Glutamate is the major excitatory neurotransmitter in the mammalian central nervous system (CNS). Neuronal excitotoxicity usually refers to the injury and death of neurons that arises from excessive activation of glutamate receptors, which lead to a number of deleterious consequences, including impairment of calcium buffering, generation of free radicals, activation of the mitochondrial permeability transition, and secondary excitotoxicity (15,16). Recent studies show that excitotoxicity is associated with a variety of neuropathological conditions and it has been suggested that excitotoxicity is a common pathogenic pathway in neurodegenerative diseases (16). 

Human epidemiological studies reported a relationship between occupational exposure to OP and neurological and neurobehavioral deficits such as impairments of memory and cognition (17,18). An increased incidence of Alzheimer’s disease (AD) has been reported among agricultural workers exposed to OP (19). Areas with high pesticide use have increased risk for AD (20). This finding has been confirmed by an experimental study. Acute and long-term exposure of cultured basal forebrain cholinergic neurons to CPF induced dose-dependent cytotoxicity (21). Repeated exposures to low-level CPF produced memory deficit in rats which was similar to AD (22,23). Although several mechanisms have been proposed for OP induced neurodegeneration, the complete mechanisms are still unknown (3). 

The aim of the present study was to compare the cytotoxic effects of CPF on cultured cerebellar granule neurons (CGNs) at different stages of development. In addition, we investigated the effects of sub-lethal concentrations of CPF on the sensitization of CGNs to glutamateinduced excitotoxicity, an involved mechanism in neurodegenerative diseases such as Alzheimer’s and Parkinson’s. 

## Materials and Methods

This study was an *in vitro* experimental study performed on mice cultured CGNs. 

### Materials

Dulbeccoʼs modified Eagleʼs medium (DMEM), heat inactivated fetal bovine serum (FBS), penicillin-streptomycin (10000 U/ml), and trypsin (0.25%) were purchased from Gibco (USA). Glutamic acid, glycine, and KCl were purchased from Merck Millipore (Germany). Cytosine arabinofuranoside (Ara-C), poly-D-lysine (PDL), CPF, and all other materials were purchased from Sigma (St. Louis, USA). 

### Primary culture of cerebellar granule neurons

CGCs were obtained from the brains of 6-7-day-old mice (BALB/c) and cultured as described previously (24). Dissected cerebella from early postnatal mice were digested with trypsin and triturated to obtain a single cell suspension. Then, the cells were seeded at a density of 1×10^6^cell/ml on PDL coated cell culture plates in DMEM that contained 10% FBS, 4.5 g/l glucose, 25 mM KCl, insulin (100 mU/L), penicillin and streptomycin 1% (v/v). Cells were maintained at 37˚C in a humidified atmosphere with 5% CO_2_. Ara-C was added to the cell culture medium at a final concentration of 20 µM to inhibit non-neuronal cell growth. This was performed 48 hours after cell plating. The culture medium was not changed during the culture period. After 7 days *in vitro* (DIV7), more than 95% of cultured cells were detected as neurons, as characterized by MAP2 protein immunostaining. All experiments were performed on DIV7 for mature neurons and DIV2 for immature neurons (25). The procedures were approved by the Medical Ethics Committee at Tarbiat Modares University.

### Toxicity of chlorpyrifos at different developmental stages

We assessed the toxicity of CPF on immature and mature CGNs, and during neuronal development. CGNs were plated on PDL-coated 96well culture plates. DIV2 immature neurons or DIV7 mature neurons were exposed to different concentrations of CPF (1-1000 µM). We measured cell viability after 48 hours using the MTT assay. In order to assess the toxicity of CPF on developing neurons, the DIV2 CGNs were exposed to different concentrations of CPF (1-1000 µM) for five days, after which cell viability was measured at DIV7 by the MTT assay. The stock solution of CPF was prepared in Dimethyl solfoxide (DMSO) and the final concentration of DMSO in the culture medium was 1% or less. 

### Toxicity of glutamate in immature and mature neurons

In order to assess the cytotoxic effect of glutamate on immature and mature neurons, both the DIV2 and DIV7 CGNs were incubated with different concentrations of glutamate (10-600 µM) for 48 hours. For each concentration of glutamate, 10 µM of glycine was added to the medium, followed by assessment of cell viability using the MTT assay after the incubation period. 

### Effect of chlorpyrifos on glutamate toxicity

We investigated the interaction between the cytotoxic effects of CPF and glutamate on mature neurons by exposing CGNs to different concentrations of glutamate (10-600 µM) combined with sub-lethal concentrations of CPF (10 and 100 µM) at DIV7. After 48 hours cell survival was assessed by the MTT assay. 

### Cell viability assay

The MTT assay was used to investigate cell viability. In this assay, the yellow tetrazolium salt (MTT) is reduced to purple formazan dye by mitochondrial dehydrogenases in live cells. The culture medium was removed and replaced with a medium that contained 0.5 mg/ml MTT reagent. After 4 hours of incubation, the medium was replaced with 100 µl DMSO to dissolve the formazan crystals, after which the absorbance was measured at a wavelength of 570 nm and a 630 nm reference wavelength. Results were expressed as percentage of control. 

### Measurement of reactive oxygen species

The CGNs were seeded on PDL-coated 96well plates (1×10^5^cells/).Both mature and immature CGNs were incubated at different concentrations of CPF (1-500 µM). ROS production was measured after 6, 24 and 48 hours. In order to measure ROS production following glutamate treatment, the mature neurons were exposed to 10-600 µM glutamate for 24 hours. The combination of various concentrations of glutamate with mature neurons incubated at 10 or 100 µM was used to investigate the effect of CPF on glutamate induced ROS production. ROS production was measured after 24 and 48 hours. ROS generation in cells was evaluated with 2´, 7´-dichlorodihydrofluorescein diacetate (DCFH-DA). DCFH-DA crosses cell membranes and is hydrolyzed by intracellular esterases to nonfluorescent DCFH, which is often used as an indicator of ROS. In the presence of ROS, DCFH is oxidized to highly fluorescent dichlorofluorescein (DCF) (26). After the incubation time, the medium was replaced with 10 μM DCFH-DA and medium. The medium was removed 15 minutes after incubation. Then, the cells were rinsed twice with Ca^2+^-free PBS. We used a fluorescence microplate reader (Biotek, USA) to measure fluorescent intensity at an excitation wavelength of 488 nm and emission wavelength of 525 nm. 

### Acetylcholinesterase activity assay

The CGNs were seeded on PDL-coated sixwell plates (1×10^6^cell).Both mature and immature CGNs were incubated at different concentrations of CPF (1-500 µM). Subsequently, we measured AChE activity using the acetylthiocholine iodide (ATCh) substratebased colorimetric method as described by Ellman et al. (27). Briefly, Ellman reagent (100 μl) that consisted of 100 mM NaHPO_4_ (pH=8.0),ATCh 75 mM (substrate), and 10 mM dithio nitrobenzoic acid (DTNB) at a ratio of 100:2:5 was transferred to the 96-well microplate. Next, 50 μl of cell lysate was added to the reaction mixture as an enzyme source. Absorbance was monitored at 405 nm for 10 minutes, then the reaction rate was calculated. A blank that contained all components except for the cell lysate was run in parallel with the sample in order to omit the spontaneous, non-enzymatic breakdown of ATCh. The AChE activity was calculated using the extinction coefficient of the TNB ion (ε=13600 µM^-1^.cm^-1^).The Bradford method was used to measure the amount of protein. 

### Statistical analysis

Each data is presented as the mean ± SEM of three separate experiments. The statistical analysis was performed by Graph Pad Prism5 software. Statistical differences were estimated using oneway ANOVA followed by the Newman-Keuls multiple comparison test. Two-way ANOVA with the Bonferroni post-test was used for statistical analysis of the combined effect of glutamate and CPF. A P value of 0.05 or less was considered statistically significant. 

## Results

### Toxicity of chlorpyrifos and glutamate at different stages of development

**Fig.1:**
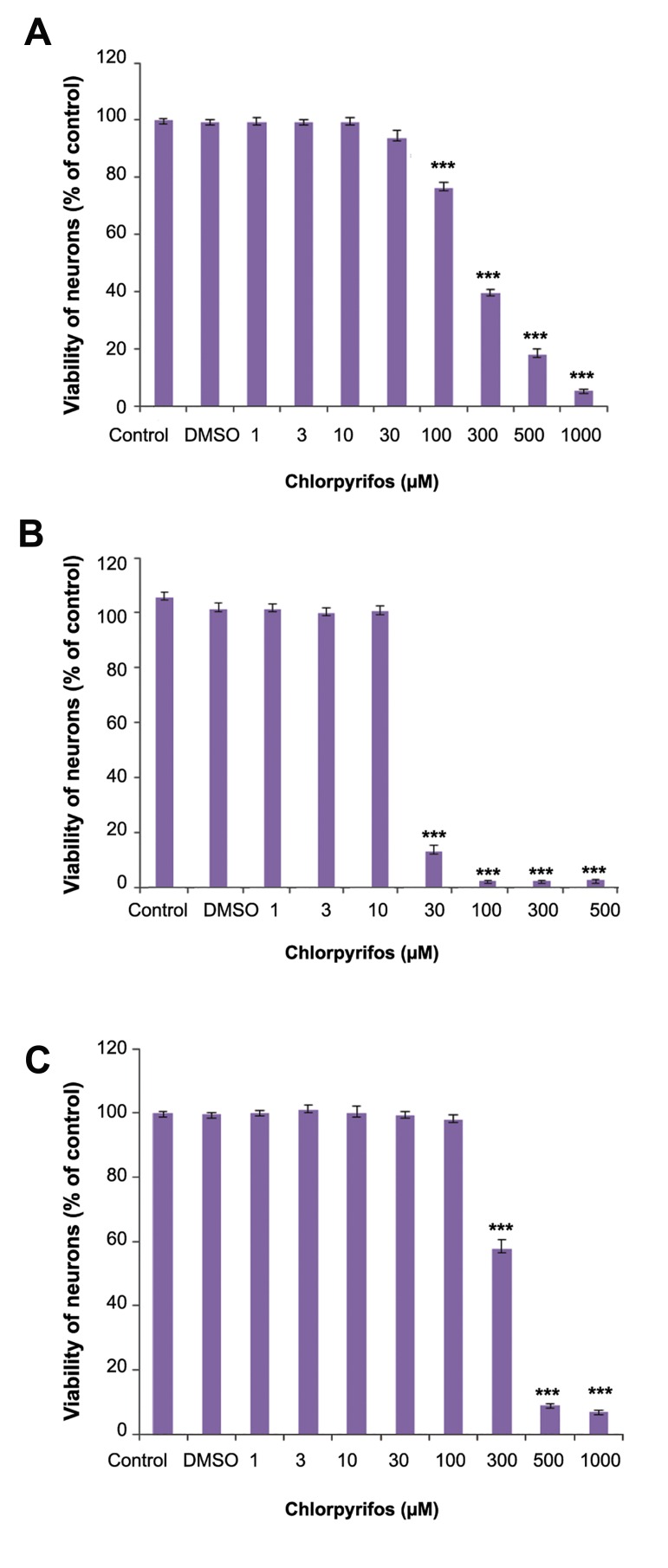
Cytotoxic effects of clorpyrifos (CPF) on cerebellar granule neurons (CGNs) at different developmental stages. A. Immature neuron, B. Developing neuron, and C. Mature neuron. ***; Significant difference versus control (P<0.001) and DMSO; Dimethyl solfoxide.

**Fig.2:**
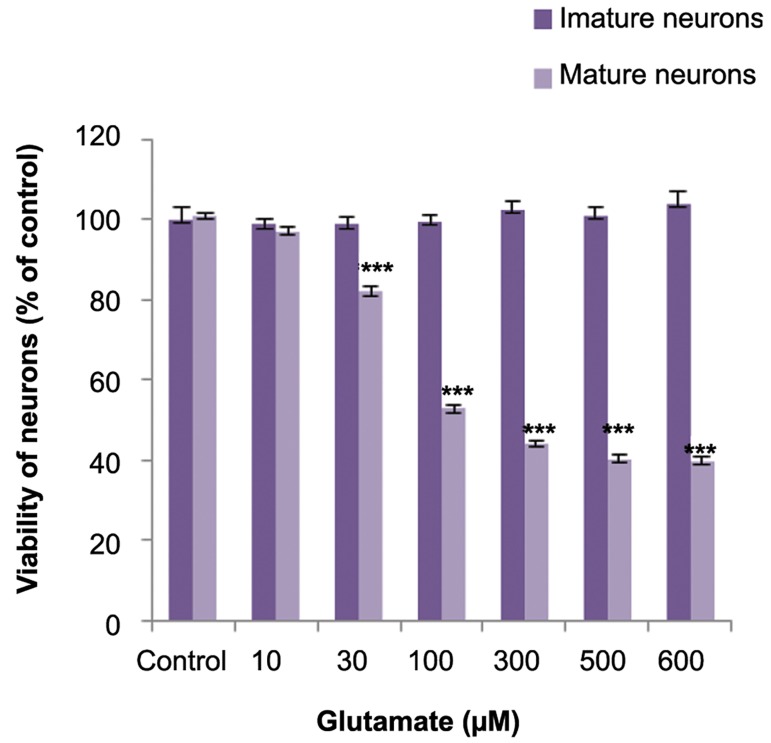
Effects of glutamate on viability of immature and mature cerebellar granule neurons (CGNs). ***; Significant difference versus control (P<0.001).

Figure 1 shows the effect of CPF on the viability of immature, mature, and developing neurons. The half maximal inhibitory concentration (IC_50_) values are given in Table 1. The IC_50_ value for immature and developing neurons significantly differed from mature neurons. Possibly, the immature neurons were more sensitive to the cytotoxic effect of CPF. The effects of glutamate on viability of immature and mature neurons were assessed. Glutamate dose-dependently reduced the viability of mature neurons but did not affect the viability of immature neurons (Fig.2).

**Table 1:** IC_50_ values


IC_50_	Confidence interval

Immature neurons	229.8 *	198.3-266.2
Developing neurons	20.8 *	16.84-25.72
Mature neurons	307.1	300.6-313.8

*; Significant differences versus mature neurons (P<0.05) and IC_50_; Half maximal inhibitory concentration.

### Effect of chlorpyrifos on glutamate cytotoxicity

**Fig.3:**
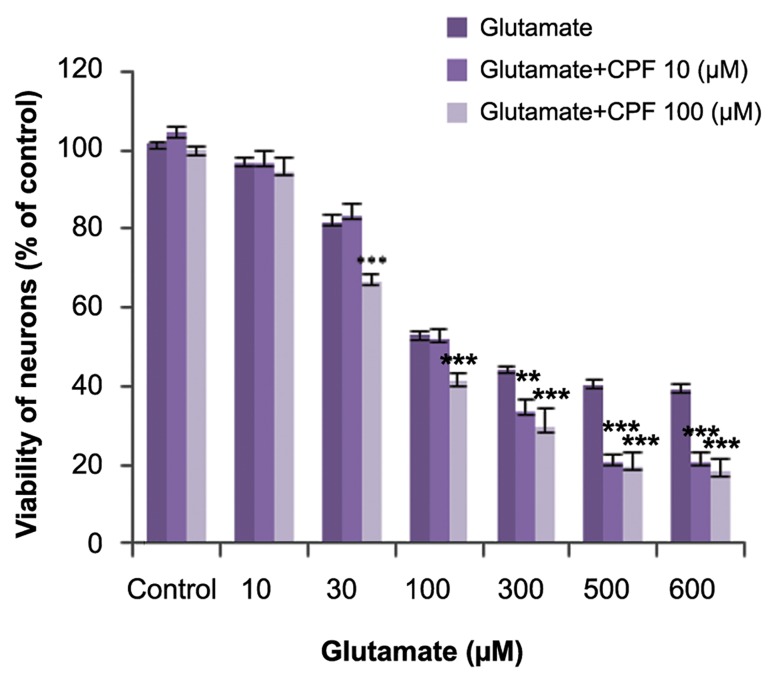
Effects of chlorpyrifos (CPF) on glutamate toxicity in mature cerebellar granule neurons (CGNs). ***; Significant difference versus control (P<0.001).

Co-incubation of sub-lethal concentrations of CPF with glutamate significantly increased the cytotoxic effect of glutamate on mature neurons (Fig.3). 

### Effect of chlorpyrifos on acetylcholinesterase activity in cultured neurons

**Fig.4:**
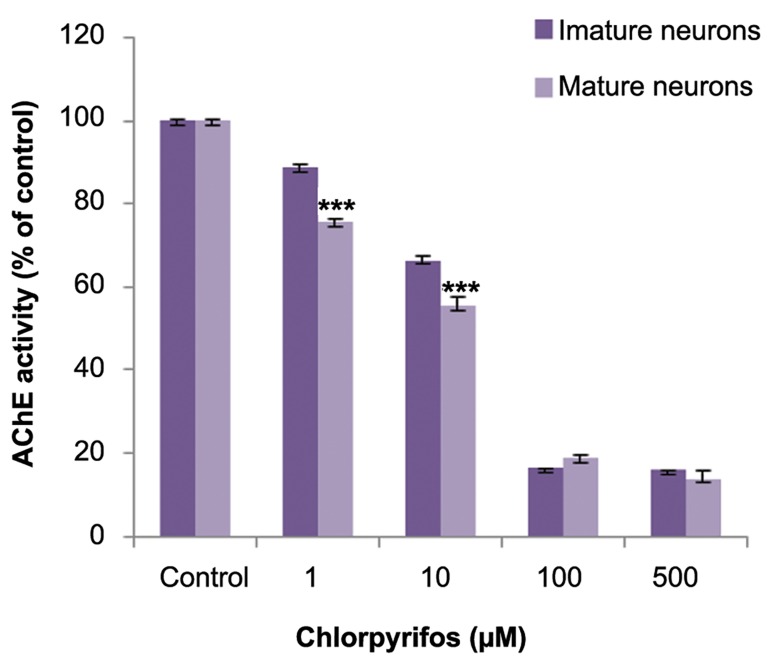
Acetylcholinesterase (AChE) inhibition following clorpyrifos (CPF) exposure in immature and mature cerebellar granule neurons (CGNs). There is a significant difference in CPF induced enzyme inhibition at low concentrations. ***; P<0.001.

The effect of CPF on AChE activity of mature and immature neurons was measured. CPF dosedependently inhibited AChE activity in both neurons. There was slight inhibition observed at low concentrations (approximately 20% at 1 µM) which increased to approximately 80% inhibition at higher concentrations (100 and 500 µM). Mature neurons showed more inhibition of AChE activity compared to immature neurons (Fig.4).

### Effect of chlorpyrifos on reactive oxygen species production

**Fig.5:**
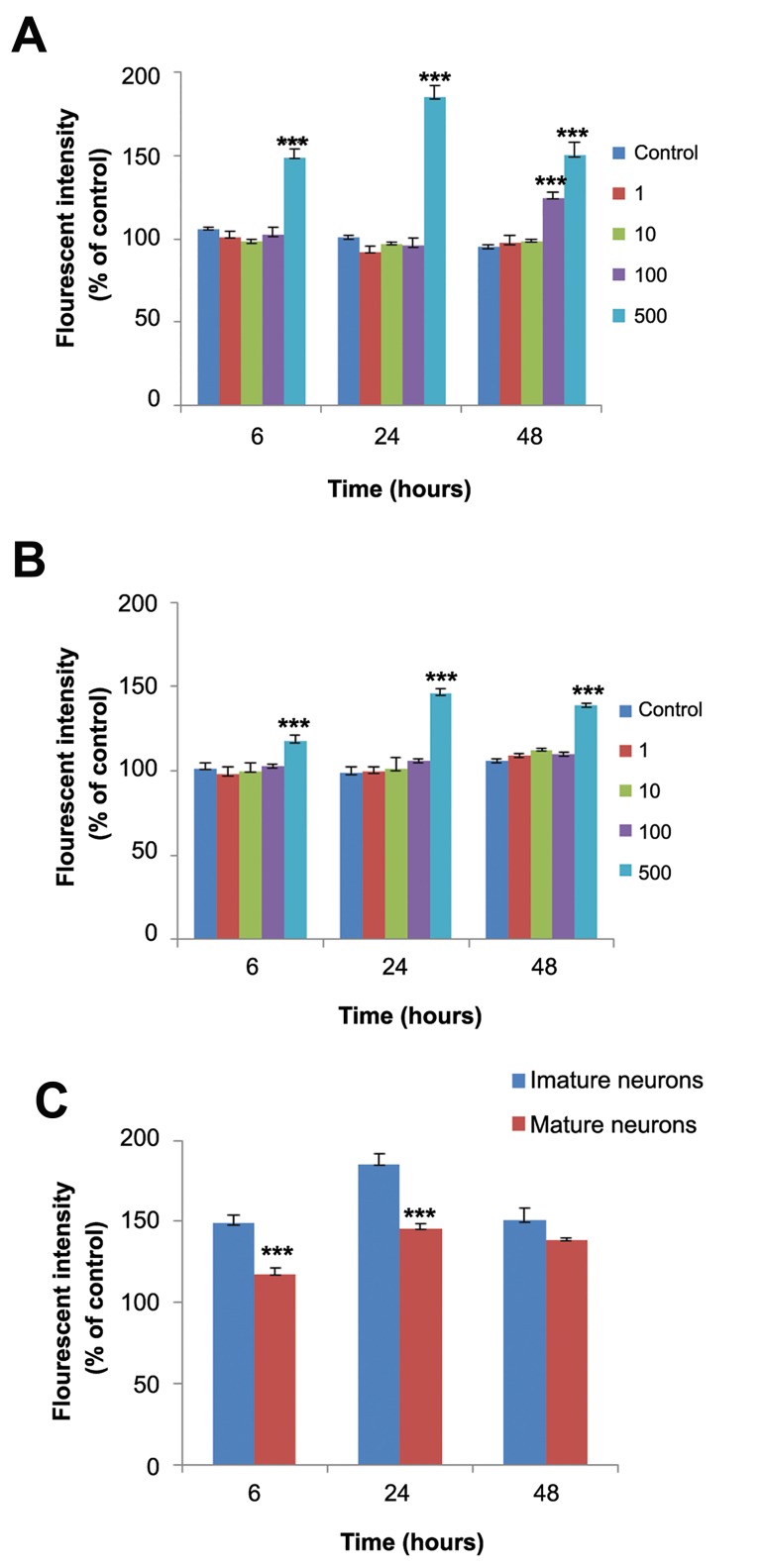
Reactive oxygen species (ROS) production following clorpyrifos (CPF) treatment at different time points. A. Immature neurons, B. Mature neurons, and C. comparison of ROS production after treatment with 500 µM CPF at different time points between immature and mature neurons. ***; P<0.001.

**Fig.6:**
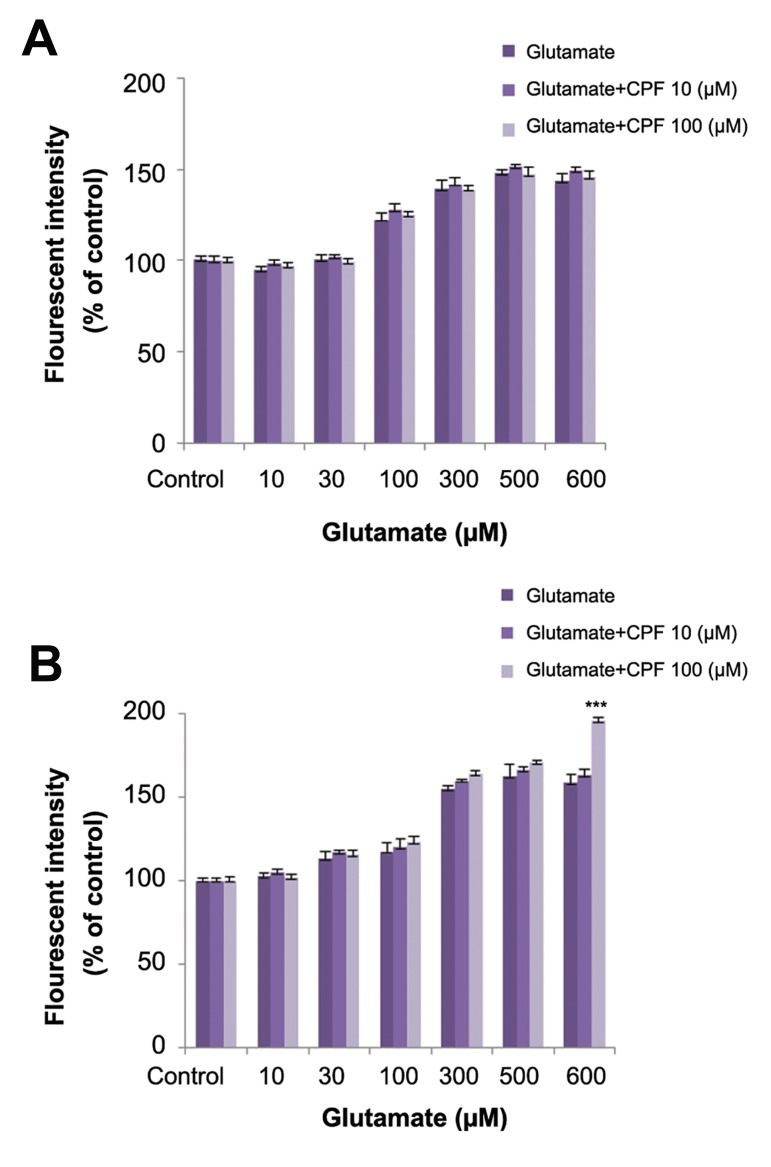
Effects of chlorpyrifos (CPF) on glutamate induced reactive oxygen species (ROS) production in mature cerebellar granule neurons (CGNs) after: A. 24-hour treatment and B. 48-hour treatment. ***; Significant difference versus control (P<0.001).

The amount of ROS production after 6, 24, and 48 hours of exposure to CPF were measured in immature and mature neurons. CPF increased ROS production only at the 500 µM concentration at all time points in the mature neurons (Fig.5A). In immature neurons, ROS production increased after exposure to 500 µm CPF at all times and after 48 hours of exposure to 100 µM CPF (Fig.5B). In addition, there was significantly higher ROS in immature neurons compared to mature neurons (Fig.5C). Co-incubation of sub-lethal concentration of CPF with different concentrations of glutamate at 24 and 48 hours had no effect on glutamate-induced ROS production except at the 600 µM concentration. Co-incubation with CPF at 100 µM and glutamate at 600 µM after 48 hours increased ROS production compared to glutamate alone (Fig.6). 

## Discussion

This study investigated CPF induced neurotoxicity on cultured CGNs at different stages of development. The results indicated that immature neurons had increased susceptibility to CPF-induced toxicity and oxidative stress than CPF-induced AChE inhibition. We observed that sub-lethal concentrations of CPF potentiated glutamate induced toxicity in mature neurons. 

The primary culture of CGNs has been well characterized as a model to study developmental neurotoxicity because the important stages of neurodevelopment (migration, differentiation, morphological, and functional maturation) occur in this cell culture (28, 29). Additionally, CGNs culture can be used as a model for studying cellular and molecular mechanisms of neural cell apoptosis, survival, neurodegeneration, and neuroprotection (30). The results of this study have indicated that immature neurons are more susceptible to CPF induced toxicity than mature neurons. The IC_50_ value for five days of exposure during development demonstrated that CPF had a greater toxic effect on immature and developing neurons which might be due to the exposure duration and increased sensitivity of immature neurons. Many studies reported the susceptibility of the developing nervous system to damage following exposure to CPF. Prenatal and early postnatal exposure to CPF resulted in brain cell loss and abnormal synaptic development which led to behavioral deficits (31, 32). Postnatal 17-day-old rats had increased susceptibility to the behavioral and biochemical toxicity of CPF when compared to 70-day-old adult rats (33).

On the other hand, the results of this study indicated that although CPF induced ROS production both in mature and immature neurons, this increase was more in immature neurons compared to mature neurons. ROS production following CPF exposure showed good correlation with the cytotoxic effects of CPF. These findings have suggested that CPF neurotoxicity may be at least partly mediated by ROS generation. Oxidative stress is more prominent in immature neurons. The expression of antioxidant systems changes with brain maturation (34) and antioxidant content such as glutathione in immature neurons is lower than mature neurons (35). Perhaps the vulnerability of immature neurons is due to a low content of antioxidant defense. 

CPF is a well-known AChE inhibitor. However, studies indicate that CPF induced neurotoxicity can be independent of AChE inhibition. Other mechanisms may be involved. The results of this study have shown that CPF toxicity did not correlate well with AChE inhibition in both immature and mature neurons. Mature neurons are less vulnerable to CPF toxicity despite greater inhibition of AChE in these neurons. CPF metabolized to the oxon metabolite by CYP450 enzymes and it has been reported that the CPF-oxon more potently inhibits AChE when compared to the parent compound. Studies showed that CYP450 enzymes exhibited less expression in developing neurons (36), therefore the immature neurons had less ability to convert CPF to CPF-oxon. Furthermore, developing neurons recovered from AChE inhibition much more rapidly than mature neurons due to greater protein synthesis (7). It was suggested that CPF rather than CPF-oxon affected cell viability in immature neurons by a mechanism other than AChE inhibition. 

One of the proposed mechanisms of CPF toxicity is excitotoxicity. We have observed in this study that glutamate is not toxic to immature neurons. This result confirmed findings of other studies where immature neurons were not sensitive to glutamate toxicity, which was due to changes in NMDA subunit compositions during development (37,38). It has been suggested that the mechanism by which CPF induces neurotoxicity is not mediated by glutamate in immature neurons; it seems that oxidative stress has a more important role in CPF induced toxicity in immature compared to mature neurons. 

In this study we co-incubated CGNs with glutamate and sub-lethal concentrations of CPF in an attempt to determine the effects of CPF on glutamate induced toxicity and cell viability. The results indicated that sub-lethal concentrations of CPF potentiated glutamate induced cytotoxicity. On the other hand, we observed that sub-lethal concentrations of CPF did not potentiate glutamate induced ROS production which suggested that mechanisms other than oxidative stress were involved in the effect of CPF. Involvement of the nicotinic receptor might be one of the possible mechanisms. It has been reported that CPF more strongly blocked nicotinic receptors than inhibition of AChE (10). Acetylcholine has been reported to protect neurons against glutamate induced toxicity through activation of nicotinic receptors (39). Possibly, blockage of nicotinic receptors by CPF might increase the sensitivity of CGNs to glutamate toxicity. Other possible mechanisms that might be involved include an increase in intracellular calcium concentration and inhibition of the cell survival signaling pathway by CPF. These possible mechanisms need further investigation. 

## Conclusion

This study indicated that CPF has different cytotoxic effects on CGNs at different stages of development which did not correlate with AChE inhibition, but correlated with ROS production. We observed that CPF potentiated glutamate induced toxicity. Excitotoxicity has been linked to various neurodegenerative diseases such as Alzheimer’s. Potentiation of glutamate toxicity by sub-lethal concentrations of CPF suggested that CPF might be a risk factor for neurodegenerative disease at a low dose exposure. 
